# Changing Trends in Publication Regarding Temporomandibular Joint Dysfunction From 1975 to 2021: A Bibliometric Analysis

**DOI:** 10.7759/cureus.46179

**Published:** 2023-09-29

**Authors:** Om C Wadhokar, Chaitanya A Kulkarni, Medhavi Joshi

**Affiliations:** 1 Musculoskeletal Physiotherapy, Dr. D.Y Patil College of Physiotherapy, Pune, IND; 2 Public Health, Jawaharlal Nehru Medical College, Datta Meghe Institute of Higher Education and Research, Wardha, IND; 3 Community Health Physiotherapy, Dr. D.Y Patil College of Physiotherapy, Pune, IND

**Keywords:** tmj, bibliometric analyis, physical therapy, neck pain, forward head, functional disability, tmd

## Abstract

Temporomandibular joint (TMJ) is a bicondylar joint. Various conditions have the same clinical presentation as TMJ dysfunction, which requires a thorough assessment of active and passive movements and palpation of the joints surrounding the joint. Many times, TMJ dysfunction occurs due to an alteration in the cervical spine. The patient complains of clicking or popping sound at the jaw and a reduced maximum mouth opening. The conditions that lead to TMJ dysfunction include bruxism, arthritis, stress, injury to the jaw, and overuse. The trend of the available literature has changed from 1972 to 2021 to assess changing trends in the publication on TMJ about authors, country, collaboration of the institutes, and the journal. We have done this bibliometric assessment. The articles were collected from the PubMed database; the keywords used were temporomandibular dysfunction (TMD), neck pain, physiotherapy, and rehabilitation. A total of 437 articles were found from 1975 to 2021, which were then converted into pictorial forms using the CiteSapce R software, and the data were interpreted. The analysis of the bibliometrics of publications on TMD, neck pain, and functional disability between the years 1975 and 2021 shows a total of 437 articles were published. The articles were from 196 sources; the highest number of publications were seen from 2014 until 2021, with the highest number of published papers by author Yoo WG and the highest number of articles published by the Journal of Physical Therapy Science. This bibliometric analysis depicts that the quantity of literature on TMD and the forward head has increased, as has the effectiveness of physical therapy interventions on the forward head in terms of correcting temporomandibular dysfunction.

## Introduction and background

Temporomandibular disorder (TMD) is one of the more common disorders occurring in females than males; a differential diagnosis is essential due to various conditions mimicking the symptoms. The standard requirements are osteoarthritis and anterior disc displacement with or without reduction. The diagnosis can be made by assessing the active-passive motion and palpating the joint [1]. Cervical spine disorder (CSD) is considered one of the significant factors predisposing to TMD. The signs and symptoms of CSD and TMD overlap significantly. Studies proved that myogenic TMD involvement, compared to bony involvement, should no longer be seen as a local disorder of the stomatognathic system [2,3].

The symptoms of the TMD include clicking, difficulty opening the mouth, and difficulty speaking, kissing, and talking. The diagnosis of TMD is made by Fonseca’s questionnaire, which is an effective tool for screening the signs and symptoms of TMD; the prevalence of TMD is 45.4% [4-6]. Most mild to moderate temporomandibular dysfunctions are managed conservatively and have good results. The commonly opted-for conservative treatments are fabricated occlusal splints, manual therapy, taping, hot and cold fermentations, and light and laser therapy. Drugs are also used in many cases to reduce pain and improve quality of life [7].

Bibliometric assessment is a qualitative statistical tool to study the already-published literature; it is applied to evaluate citation count, collaborations, institutions, journals, authors, and keyword trends in the research field. The aim of the current study was to conduct a bibliometric analysis to summarize the progress and trends of physical therapy in treating TMD. The results will indicate cited publications, co-cited journals, collaboration between countries and institutions, and authors.

## Review

Methods

Search Strategy

For data collection, the articles were searched in the PubMed database for the past 50 years, from 1972 to 2021. The data search keywords were "temporomandibular joint dysfunction", "neck pain", "physical therapy", "rehabilitation", "conventional physiotherapy", "forward head posture", and "conservative management".

Analysis Tool

CiteSpace 5.3.R4 (developed by Dr. Chaomei Chen, 2003-2021), Microsoft Excel 2016 (Microsoft Corp., Redmond, WA, USA), and IBM SPSS Statistics software version 20.0 (IBM Corp., Armonk, NY, USA) were used to analyze the data. The R software (The R Core Team, R Foundation for Statistical Computing, Vienna, Austria) was used to find the article based on the keywords used and represent the data in pictorial form based on authors, citations, collaborations of countries, institutes, sources, etc. Microsoft Excel 2016 was used to tabulate and construct a trending figure of publication quantity with years. In addition, IBM SPSS Statistics 20.0 software was used to carry out Pearson’s correlation analysis of the year and publication quantity.

Data Extraction

The relational figures and tables are obtained by data interpretation with the analysis tool. The study interprets global research reports on exercise intervention on TMD, assessment of collaboration of authors, interpretation of distribution tendency according to journals, years, and author-analysis of references and keywords

Results

**Figure 1:**
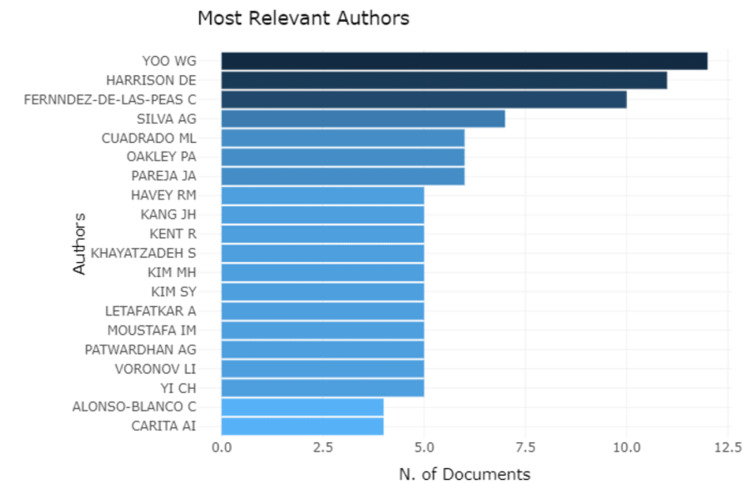
The author-wise distribution of the documents published The image has been created by the authors using the R Software.

The interpretation based on the authors shows Yoo WG, Harrison DE, and Fernindez-DE-LAS-Peas C have the highest number of publications, scoring more than 10.0. while Silva AG, Cuadrado ML, Oakley PA, and Pareja JA have publication scores between 5.0 and 7.5. Eleven authors have publications equal to 5.0, and two have publications below 5.0. The graphical representation is mentioned in Figure 1.

**Figure 2:**
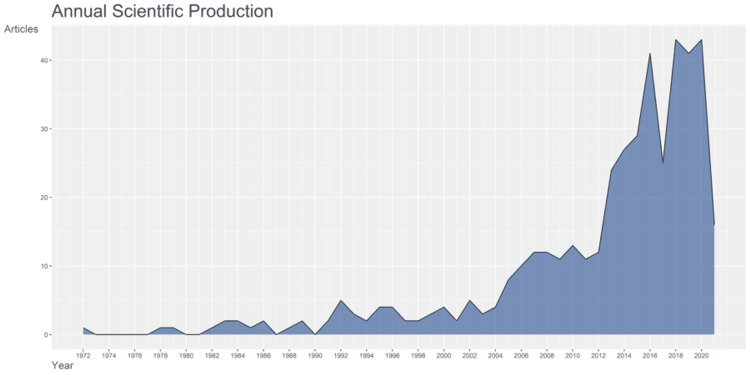
A report of the number of articles published annually from 1972 to 2020 The image has been created by the authors using the R Software.

A total of 437 references were included. The number of publications increased over the years, a significant increase from 2012 to 2020. There were below 10 articles every year until the year 2006. After that, there was considerable improvement in the rate of publication between the years 2004 and 2012. From 1972 to 1990, the number of documents published was less than three; from 1990 to 2004, there were around four to five publications per year, as shown in Figure 2.

**Figure 3:**
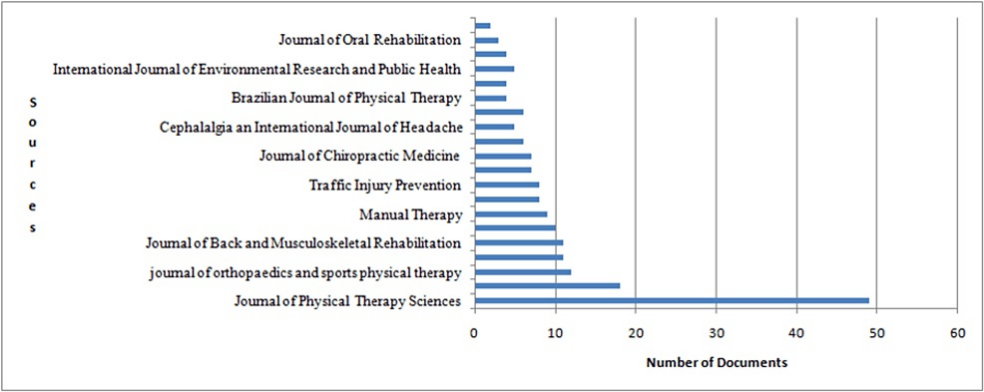
A representation of the data based on the most relevant sources of articles The image has been created by the authors using the R Software.

The Journal of Physical Therapy Science has the most relevant articles, which is nearly equal to 50. The rest of the 19 journals have an article count of less than 20, most pertinent to the keywords used for searching in the database. The pictorial representation is mentioned in Figure 3.

**Figure 4:**
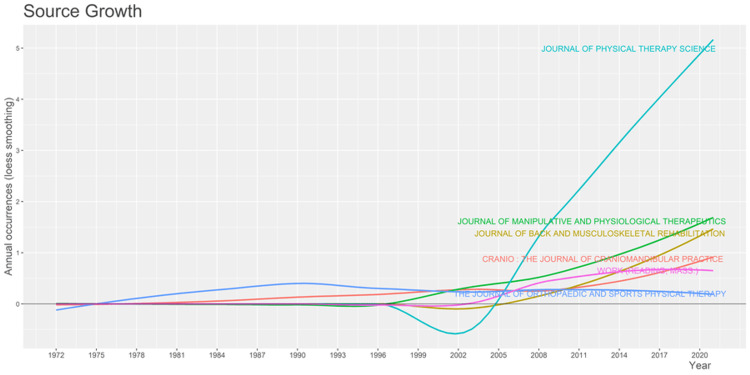
The journals that were the sources of growth for the publication of articles The image has been created by the authors using the R Software.

The sources of growth are the Journal of Manipulative and Physiological Therapeutics, the Journal of Back and Musculoskeletal Rehab, and Cranio. The annual occurrence of articles is equal to or less than two, while the Journal of Orthopedics and Sports Physical Therapy increased in publication from 1984 to 1993. After that, the number of articles published annually decreased exponentially until 2020. The Journal of Physical Therapy Science has had steady, significant, positive growth from 2005 to 2020 (Figure 4).

Temporomandibular dysfunction with orofacial pain with or without reduced jaw mobility is a common condition seen in the general population. Various factors, such as tooth clenching and grinding because of increased psychosocial stresses or direct trauma or fracture, are the main etiological factors for TMD. The studies have shown that a well-planned physical therapy protocol has improved the quality of life of patients suffering from TMD. This current analysis used articles from the past decade relating to TMD, physical therapy, and conservative management. A total of 580 articles were obtained. The data obtained were imported into the CiteSpace software, which is obtained from the database using keywords, journals, number of publications, etc. The pictorial representation of the data was obtained from the software, and interpretation was made [8-10].

Systematic information was obtained regarding the global research into exercise therapy for TMD over the last decade. This showed a significant increase in the number of publications on this topic since 2014 and continues until 2020. Before that, moderate articles were published from 2006 to 2014. While these values are the number of publications, they do not relate to the quality of the material published [11-16].

The top journals are the Journal of Physical Therapy Science (nearly 50 articles), followed by the Journal of Manipulative and Physiological Therapeutics, the Journal of Orthopedics and Sports Physical Therapy, and the Journal of Craniomandibular Practice (with nearly 12-20 articles). According to our study, the publication rate has increased significantly over the period, and the application of exercise to the cervical region has reduced the TMD significantly.

## Conclusions

This analysis shows the increasing trends in the publication on TMD and the effectiveness of conservative therapy for treating TMD. The number of collaborations between the countries has also increased. In conclusion, more studies should be done to find the most suitable intervention for TMD.
